# Bruins-in-Genomics: Evaluation of the impact of a UCLA undergraduate summer program in computational biology on participating students

**DOI:** 10.1371/journal.pone.0268861

**Published:** 2022-05-27

**Authors:** Hilary A. Coller, Stacey Beggs, Samantha Andrews, Jeff Maloy, Alec Chiu, Sriram Sankararaman, Matteo Pellegrini, Nelson Freimer, Tracy Johnson, Jeanette Papp, Eleazar Eskin, Alexander Hoffmann

**Affiliations:** 1 Department of Molecular, Cell and Developmental Biology, University of California, Los Angeles, CA, United States of America; 2 Department of Biological Chemistry, David Geffen School of Medicine, Los Angeles, CA, United States of America; 3 Bioinformatics Interdepartmental Program, University of California, Los Angeles, CA, United States of America; 4 Molecular Biology Institute, University of California, Los Angeles, CA, United States of America; 5 Institute for Quantitative and Computational Biology, University of California, Los Angeles, CA, United States of America; 6 Department of Computational Medicine, David Geffen School of Medicine, Los Angeles, CA, United States of America; 7 Department of Computer Science, University of California, Los Angeles, CA, United States of America; 8 Department of Genetics, David Geffen School of Medicine, Los Angeles, CA, United States of America; 9 Department of Psychiatry and Biobehavioral Sciences, David Geffen School of Medicine, Los Angeles, CA, United States of America; 10 Department of Microbiology, Immunology, and Molecular Genetics, Los Angeles, CA, United States of America; Sreenidhi Institute of Science and Technology, INDIA

## Abstract

Recruiting, training and retaining scientists in computational biology is necessary to develop a workforce that can lead the quantitative biology revolution. Yet, African-American/Black, Hispanic/Latinx, Native Americans, and women are severely underrepresented in computational biosciences. We established the UCLA Bruins-in-Genomics Summer Research Program to provide training and research experiences in quantitative biology and bioinformatics to undergraduate students with an emphasis on students from backgrounds underrepresented in computational biology. Program assessment was based on number of applicants, alumni surveys and comparison of post-graduate educational choices for participants and a control group of students who were accepted but declined to participate. We hypothesized that participation in the Bruins-in-Genomics program would increase the likelihood that students would pursue post-graduate education in a related field. Our surveys revealed that 75% of Bruins-in-Genomics Summer participants were enrolled in graduate school. Logistic regression analysis revealed that women who participated in the program were significantly more likely to pursue a Ph.D. than a matched control group (group x woman interaction term of *p = 0*.*005*). The Bruins-in-Genomics Summer program represents an example of how a combined didactic-research program structure can make computational biology accessible to a wide range of undergraduates and increase participation in quantitative biosciences.

## Introduction

Our nation has been challenged to add one million STEM-trained individuals to the workforce [1] because workers with these skills play a critical role in the scientific and technological innovation that drives the competitiveness of the U.S. economy. Underrepresented minority (URM) groups represent some of the fastest growing sectors of our society [2] and have the potential to be an important pool of advanced degree holders. Researchers from different backgrounds have distinct perspectives and frames of reference, and can identify unique questions. Indeed, demographically underrepresented Ph.D. students innovate at higher rates than majority students, even while the scientific community has struggled to embrace these innovators [3]. Among STEM fields, biology holds enormous potential to impact the economy, public health, energy production, and other areas. The explosion of quantitative data in biology has been accompanied by an increasing need for computational and quantitative methods to collect, process and analyze data, and to understand and predict the behavior of complex systems [4, 5].

Despite these needs, students from Black/African-American, Hispanic/Latinx and Native-American backgrounds are severely underrepresented in graduate programs in STEM fields [6]. Although women are more likely than men to complete college and attend graduate school [7], they are vastly underrepresented in the computational sciences [8]. This talent underutilization compromises our nation’s ability to continue its role as a leader in technology and innovation [1].

It is critical that life scientists receive quantitative training that will allow them to fully participate in the bio-data science revolution [4, 9, 10]. Multiple studies have highlighted the need for undergraduate students to develop quantitative skills and learn to apply them to biological problems [11–13]. Unfortunately, training of life scientists in quantitative disciplines has not adequately addressed this need [5, 10, 14]. Previous work has suggested that socialization patterns contribute to underrepresented and female students exhibiting decreased interest, enjoyment, and academic self-confidence in quantitative fields [15]. Moreover, recent work suggests that persistence in fields is critically tied to a student’s sense of scientific identity [16]. There is a strong need for interventions that build skills, self-efficacy, and quantitative science identity to narrow the representation gap in quantitative biology.

There is substantial evidence that providing research opportunities to undergraduates has a significant impact on their careers. Undergraduates who participate in research are more likely to persist in STEM majors, identify as scientists, maintain interest in STEM-related careers, and attend a graduate program in the sciences [17–19]. Attributes of undergraduate research experiences important for their success are opportunities for inquiry-based learning [20, 21], learning within a community [22, 23], and strong faculty mentorship [24–26]. Working closely with faculty on research can foster a sense of scientific identity [27] and help promote equitable outcomes for students from diverse backgrounds [28, 29] and is a strong predictor of future graduate study [26, 30].

In addition to research experiences, other critical factors can contribute to the likelihood that a program will have short and long-term positive outcomes for its participants [16, 31, 32]. Successful undergraduate research programs often include extensive mentorship support and professional socialization components such as the opportunity to present original research to fellow scientists to increase student interest, identity, and efficacy [33]. One particularly successful example is the Meyerhoff Scholars Program which is designed to address student isolation and lack of support with a plan that stresses academic and social integration, advising, monitoring, and knowledge and skill development [34].

We designed the UCLA Bruins-in-Genomics (BIG) Summer undergraduate research program, launched in 2016, to address the specific need for a more robust and diverse pipeline of computational and quantitative biologists. The aim of BIG Summer is to provide a diverse group of undergraduates with training in quantitative analysis of genomic data, an inquiry-based research experience, a sense of community and scientific identity, mentorship from a faculty member, and support for professional development. We anticipated that a program with these elements would have a strong impact on the students’ career paths, and we specifically hypothesized that program participants would be more likely than controls to enter graduate programs in bioinformatics and related fields. Here we describe the features of the BIG Summer program and our analyses of its impacts on the participating students. We found that among the students who had received their bachelor’s degree and for whom we have data on their career paths, 75% of all students and 69% of URM students had entered post-undergraduate degree programs. A logistic regression analysis revealed that for women in particular, participating in the program was associated with a higher likelihood of enrolling in a PhD program.

## Conceptual framework

In developing and assessing the BIG Summer undergraduate research program, we relied on the Tripartite Integration Model of Social Influences (TIMSI), which describes three fundamental developmental processes that promote student integration into STEM careers [35]. Guided by Kelman’s social influence theory, TIMSI emphasizes the importance of self-efficacy, scientific identity, and scientific value endorsement in promoting socialization into the scientific community, which in turn promotes distal outcomes such as academic persistence and pursuit of scientific careers [36].

Within the context of undergraduate STEM education, specific training environments, co-curricular opportunities, and members of the scientific community such as faculty mentors can act as social influence agents that affect the development of these three key social influence processes [37]. While some undergraduate research programs emphasize the self-efficacy (skill-building) or scientific identity components of scientific training, the TIMSI would predict that this focus may promote STEM integration for students who already identify strongly with the scientific community and/or internalize scientific values. A large body of literature suggests that this focus may disproportionately exclude women and underrepresented students from STEM fields [38]. Indeed, it is becoming increasingly clear that the most successful undergraduate research experience programs not only promote scientific self-efficacy, but also encourage project ownership and mentor support in substantial ways, thus promoting deeper integration of participation into the scientific community through multiple social influence processes [39].

**Fig 1:**
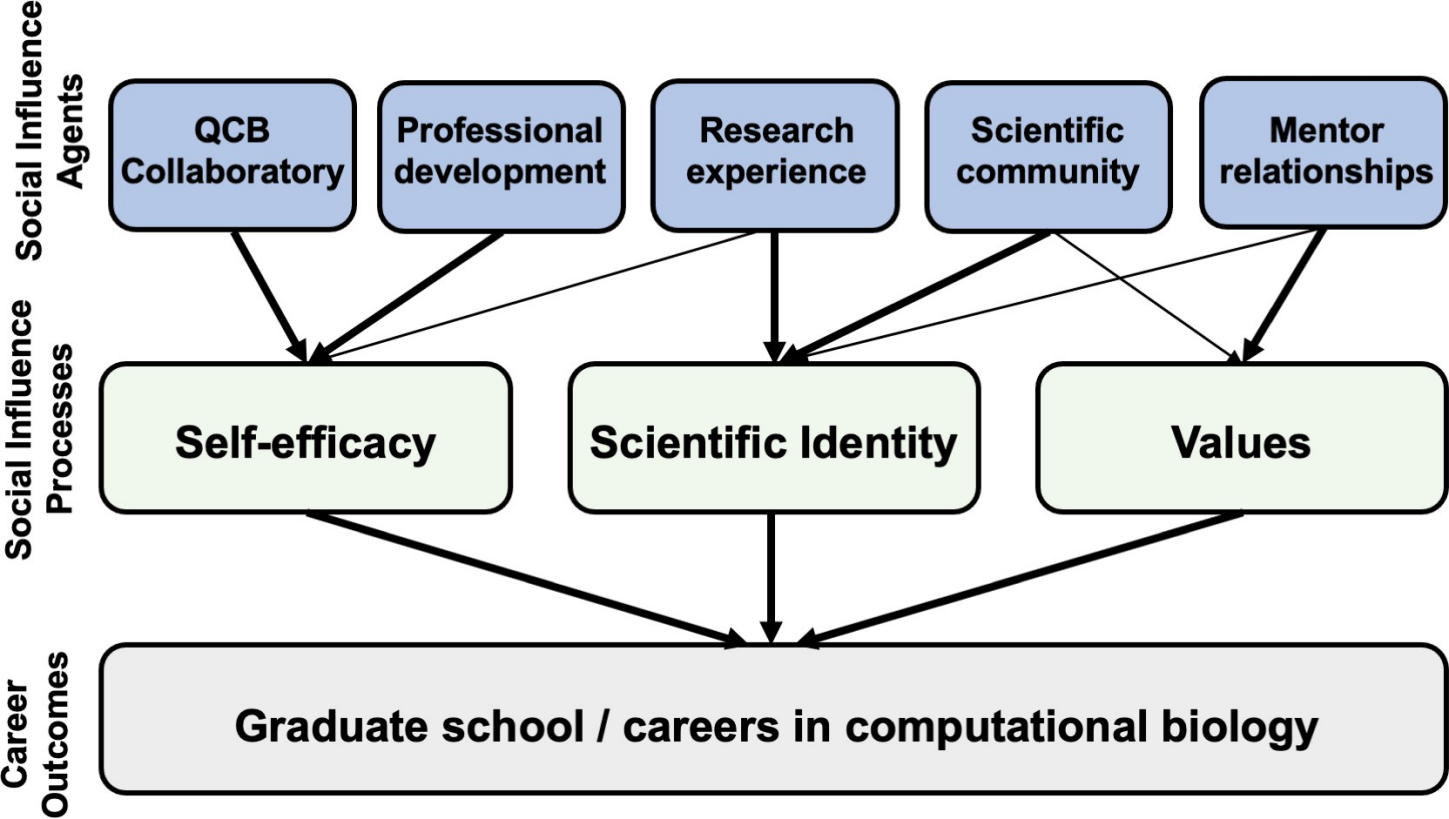
Schematic of the components and outcomes of the Bruins-in-Genomics Summer undergraduate research program. The social influence agents, social influence processes and their relationship to career outcomes are depicted.

The components of the BIG Summer program were developed in a way that positions them as social influence agents that simultaneously target the fundamental social influence processes predicted to be important for positive longitudinal outcomes for a diverse group of students (Fig 1). These components consist of practical skills-based workshops to build self-efficacy in the area of quantitative biology, authentic research experiences and structured professional development opportunities to build scientific identity, and scaffolded intensive mentor-mentee relationships to foster internalization of scientific values. Informed by the TIMSI, we predicted that the inclusion of these components, which will be described in further detail below, would encourage integration of BIG Summer participants into the quantitative biology community and therefore promote aspirations to obtain a graduate degree in quantitative biology.

## Methods

All studies were approved by the University of California Institutional Research Board as having minimal risk. The IRB determined that it was not necessary to obtain signed informed consent from participants. The IRB determined that the recruitment email could serve as the informed consent form. All relevant federal and institutional guidelines were followed. Graduates of the BIG Summer program were contacted by email and asked to participate in surveys administered through Google forms. Of the 187 alumni of the program that were emailed, 70 responded. Emails to the students explained that the data generated would be part of our research on the summer program and could be published in research journals. Student alumni were told that they could address any questions about the survey to program administrators. The survey that was administered via Google docs is provided as a pdf as Supplementary Information Document 1 in S1 File. Students could choose to not answer the survey at all or skip individual questions. The survey was taken via secure internet forms. Spreadsheets with identifiable data were maintained on encrypted, password-protected and firewall-protected computers.

To assess the current status of BIG Summer alumni, web-based searches were performed through Google, LinkedIn and other publicly accessible websites. These searches resulted in information about whether the students had matriculated in a graduate program and if so, in what field. The data abstraction document is provided as Supplementary Information Document 2 in S1 File. The same web-based searches were performed on a control group of students who applied to the BIG Summer program and were accepted into the program, but chose not to enroll. The same procedures for internet searches were applied consistently for all students in the participant and control groups. We applied the chi-square method to test for differences between the control group and the group of students who attended BIG Summer. A logistic regression model was generated with categories for whether the student participated in the program, gender, URM status, and whether the student pursued a PhD for graduate study. Logistic regression analysis was performed using scripts written in Python. Student data were loaded as a dataframe with the pandas module. The statsmodels Python modules were used to generate a logistic model of the data.

## Results

BIG Summer is an 8-10-week program that provides undergraduate students an opportunity to gain skills in performing research in computational biology. The program has five components (Fig 1): (1) A didactic component provided by the UCLA Institute for Quantitative and Computational Biology (QCBio) Collaboratory which provides workshops in a classroom learning setting on the practical bioinformatic skills required for the analysis of Next Generation Sequencing data, (2) Professional development workshops provided by diverse presenters, (3) An authentic research experience in UCLA laboratories designed and guided by mentoring faculty and their graduate student and postdoctoral trainees, (4) A scientific community with enriching events such as journal clubs, research seminars, and social events, and (5) Mentoring relationships provided by mentoring faculty and teaching assistants.

**Fig 2:**
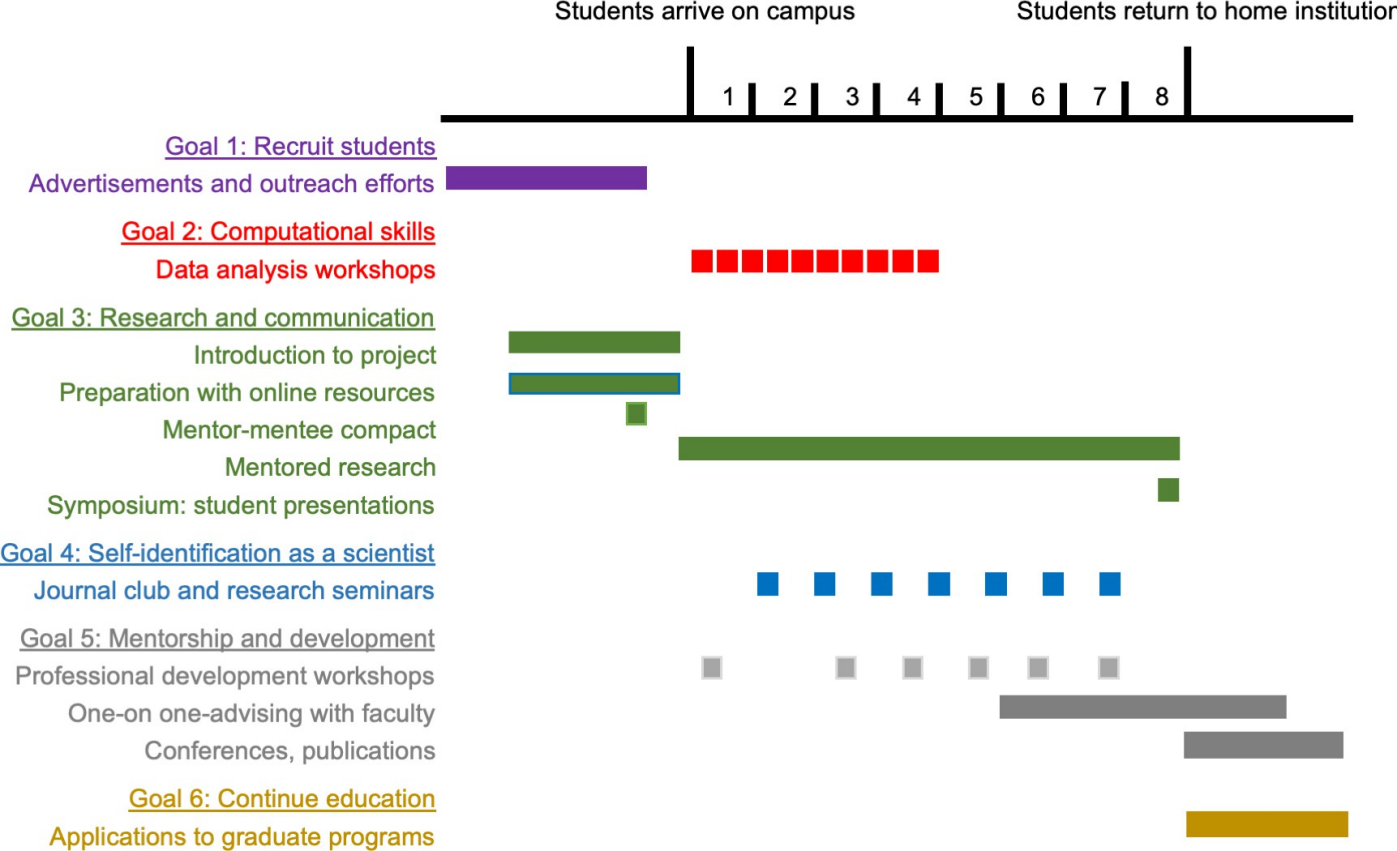
Gantt chart to indicate the timeline of various program activities grouped around the most relevant program goal that they aim to support. In addition to the categories shown here, many activities support multiple program goals.

The goals of the program, as described in detail below, are focused on the undergraduate student participants, which on their own justify the investment in faculty time and funds. However, over the past years it has become clear that the outcomes of the program are broader. Faculty mentors report that undergraduate program participants are making important contributions to their research projects. Postdoctoral and doctoral trainees report improved skills in mentoring and project management, while faculty and departments note an improved institutional climate when community members engage in a shared focus on furthering diversity and mentoring. These latter outcomes help to sustain enthusiasm among the available faculty pool to serve as mentors in BIG Summer. The five components of the program were designed to address six program goals, as described below, through a variety of activities (Fig 2).

### Program goal 1. Recruit a diverse cohort of students interested in computational biology

The BIG Summer program is advertised through in-person and electronic advertising beginning in the fall of the previous calendar year (Fig 2). Affiliated faculty announce the program in classrooms and at invited lectures. Websites and Twitter feeds for UCLA Departments and QCBio share information about the program. Program representatives recruit students from national conferences. Emails advertising the program are sent to leaders in quantitative biology and faculty who have written recommendation letters for applicants in previous years. Information about the program is disseminated to faculty at partner institutions, such as Hispanic-serving institutions Cal State University Los Angeles and Cal State University Northridge, Native American-serving institutions Fort Lewis College and Heritage University, and Historically Black Colleges and Universities Florida A&M, Morehouse, Spelman, and Fisk. We also recruit at undergraduate STEM research conferences such as the Annual Biomedical Research Conference for Minority Students (ABRCMS) and the Annual Conference of the Society for Advancement of Chicanos/Hispanics and Native Americans in Science (SACNAS).

**Fig 3:**
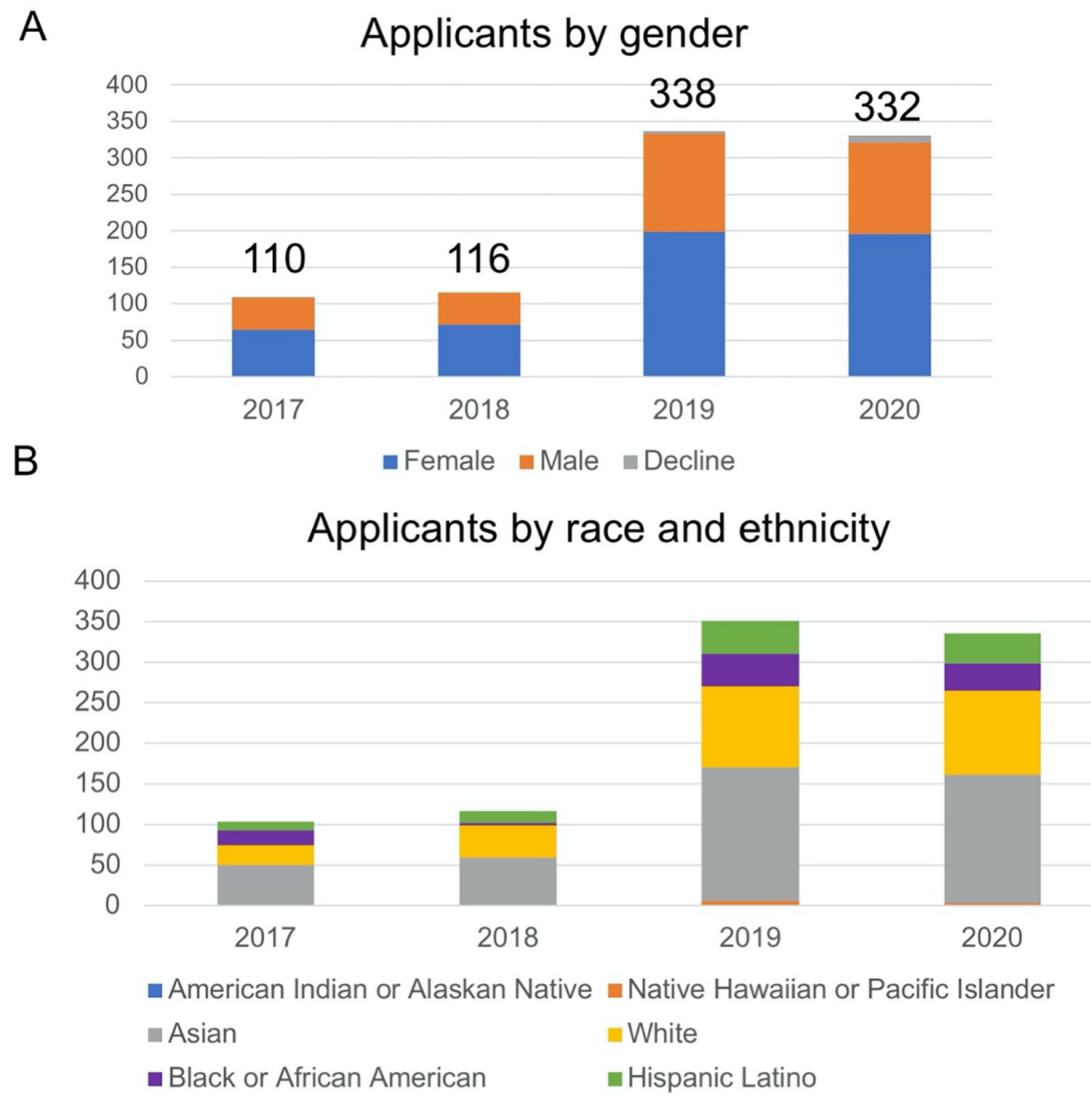
Applicants over time. (A) The number of applicants to the Bruins-in-Genomics Summer Program from 2017 to 2020 is plotted. Students are broken down by gender. (B) The number of applicants that categorized themselves into different race and ethnicity categories is plotted for 2017–2020. If applicants indicated multiple categories, they are included twice.

In 2017 and 2018, the BIG Summer program received 110 and 116 applicants, respectively. In subsequent years the number tripled, reflecting improved recruitment efforts and program reputation (Fig 3A). In each year since the inception of BIG Summer, the applicant pool has been greater than 50% women. In 2020, we received 195 applications from women among 321 total applications (Fig 3A). From 2017–2020, the program received 197 URM applicants out of 893 total applicants (22%) (Fig 3B). For comparison, the fraction of all undergraduate students who are URMs in all fields of biology was 17% in 2013, and the fraction of students who enrolled in a PhD program in all fields of biology who are URMs is 14% [40]. This reflects the success of our vigorous recruitment of talented URM students.

**Fig 4:**
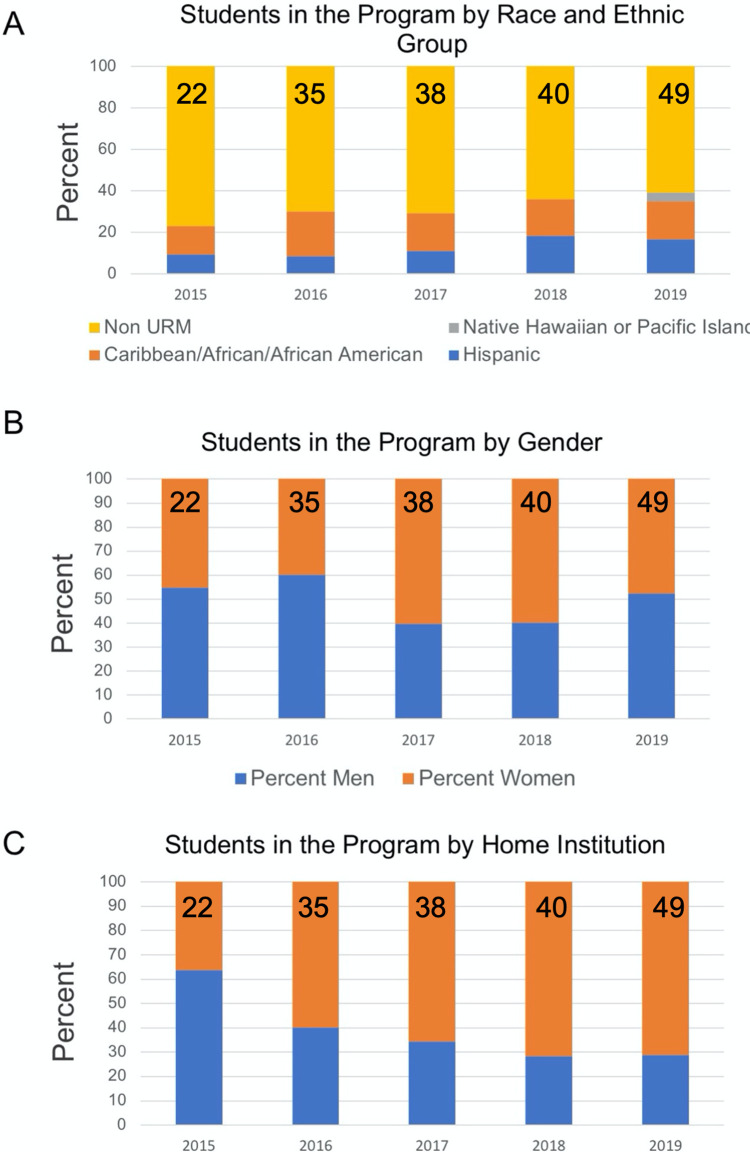
Characteristics of the participants in the Bruins-in-Genomics Summer Program over time. (A) The number of participating students in different racial and ethnic groups for each summer cohort is plotted. (B) The number of participating students is plotted for each summer cohort by gender. (C) The number of UCLA and non-UCLA students in each summer cohort is plotted.

With this advertising plan and applicant pool, the BIG Summer Program was able to establish diverse cohorts of students (Fig 4A). In 2019, 19 out of 49 (38%) students were URMs. Roughly 50% of the students in the program have been women (Fig 4B).

While in the first year of the program many of the students came from UCLA (63%), the number of students from other institutions increased over time as the reputation of the program spread throughout the country (Fig 4C). In 2019, 72% of the students were from outside UCLA.

Applicants to the program were consistently very strong academically. The average GPA of the applicants was 3.6 each year of the program. Most of the applicants are majoring in biology, bioinformatics, computer science or related fields.

### Program objective 2. Promote student learning objectives in computer programming skills and data analysis skills

Scientific self-efficacy is one of the cornerstones of the TIMSI and has been demonstrated to be a particularly important component of scientific integration for undergraduate students [35]. Therefore, a key objective of our program was to improve the self-efficacy of participants in the realm of quantitative skills.

#### Student learning objective A. To develop competence with computer programming skills

The first two weeks of the BIG Summer program involves didactic training with hands-on workshops in Next Generation Sequencing data analysis taught through QCBio (Fig 2). In the Collaboratory, a partnership between QCBio and the UCLA Division of Life Sciences, postdoctoral fellows in UCLA research laboratories teach interactive workshops on next generation sequencing analysis. The classes taught in the first two weeks provide students with a concrete set of skills to ensure they have a valuable skillset to bring to their host lab, thus increasing their confidence in their ability to contribute to the research.

These classes also introduce students to their fellow BIG Summer students, and foster community building within the summer cohort. The Collaboratory workshops provide students with training in the Unix operating system and programming in Python and R. (Student learning objectives are summarized in Supplementary Information Document 3 in S1 File).

#### Student learning objective B. To develop competence with skills needed to process, and analyze next generation sequencing data

The Collaboratory workshops provide students with hands-on training in pipelines for next generation sequencing analysis including pipelines for RNA-seq, single cell RNA-seq, and variant discovery. In addition to preparing students for research experiences, these intensive skills-focused courses provide students with experience in quantitative techniques that are broadly applicable to careers in quantitative biology fields. In the follow-up survey administered to BIG Summer alumni, 100% of respondents indicated that the BIG Summer program “Taught [them] skills that are useful to [their] career and/or studies” (n = 70) (Table 1).

**Table 1:** Bruins in Genomics Summer survey results.

	Yes	No	I don’t know
How did BIG Summer impact you?			
Taught me skills that are useful to my career and/or studies	70	0	0
Influenced my research interests	68	1	1
Introduced me to new scientific areas	66	4	0
Made connections that are useful to my career	62	6	2
Inspired me to go to graduate school	46	14	10
Helped me choose the right graduate program	42	15	13
	Yes	No	Working on it
Did your research result in a publication	13	49	49
	Yes	No	
Have you recommended BIG Summer to other students?	64	6	0

### Program objective 3. Promote student learning objectives in inquiry-driven research and scientific communication

As discussed above, prior research has demonstrated the efficacy of authentic research experiences in promoting the development of scientific identity in students [17–19]. By participating in inquiry-driven research in the laboratories of UCLA faculty mentors, BIG Summer participants were encouraged to develop ownership of a quantitative biology project. Additionally, the scaffolded structure of faculty and daily mentorship opportunities for participants was developed to expose students to scientific role models and promote the internalization of scientific community values.

#### Student learning objective C: To develop skills to critically read, interpret and evaluate primary papers in the quantitative biology literature

To help the students achieve the learning goal of increased fluency in evaluating the primary literature, students participate in a mentored, weekly journal club. The faculty leaders assign a paper each week, and work with each group of students to develop the presentation and lead a discussion (Fig 2). These journal club sessions provide an opportunity for students to gain experience critically analyzing the literature.

#### Student learning objective D: To develop research skills through inquiry-driven research projects in individual laboratories

Students perform research projects in the laboratories of faculty mentors starting in week 3 of the program (Fig 2). Faculty mentors are UCLA professors performing research in the area of quantitative, systems and computational biology. When possible, students are assigned to projects in pairs. We have found that pairing students results in peer-to-peer learning that the students find enriching and allows them to consolidate their skills by teaching each other. In addition to a faculty mentor, students are also assigned a daily mentor. Daily mentors are graduate students, postdoctoral fellows or research scientists working in the laboratory of the faculty mentor. Daily mentors work directly with the students on their projects. The faculty mentors, daily mentors and the students work together to select a project of interest to the students that relates to the research in the faculty mentor’s laboratory. Projects build upon the skills that the students learned in their Collaboratory workshops. Students work together with their partners and meet regularly with their day-to-day mentor, with guidance from the faculty mentor, on their project. In their research projects, students develop skills needed to read and analyze primary papers in the quantitative biology literature. They gain skills in developing a research question and formulating a specific hypothesis. They learn to design experiments to test their hypotheses and to collect data to test their hypotheses. Students also learn to analyze the results of their experiments to determine whether their hypotheses are correct, and to use these data to develop the next experiment. Over the course of the summer, the students take on more and more responsibility. As part of their research in individual UCLA laboratories, students attend and participate in lab meetings. In the survey administered to alumni, students overwhelmingly indicated that their research experiences through BIG Summer influenced their research interests (Table 1). Ninety-four percent of BIG Summer alumni who responded to our follow-up survey reported that their summer research experience introduced them to new scientific areas (Table 1).

#### Student learning objective E: To develop skills communicating research findings in written and oral formats

Toward the end of the summer, faculty mentors and daily mentors support the students as they write an abstract that summarizes the problem they were investigating, their specific hypothesis, their results, and their conclusions (Fig 2). The program culminates in a poster session where each of the student pairs presents a poster and describes their research to members of the UCLA community. These poster sessions are well-attended by members of the participating labs across UCLA, and give students a chance to interact as fellow scientists with established researchers, contributing to the students’ process of self-identification as a scientist. In 2020 and 2021, the poster session occurred through Zoom and the posters were replaced with oral presentations. Presentation prizes are awarded based on evaluation at the presentation session, and research excellence prizes are awarded based on faculty mentors’ recommendations. These summer research experiences resulted in authorship on publications for many participants. In response to our survey, nearly 20% of respondents indicated that their work had been published, and an additional 11% said they were in the process of preparing a manuscript for publication (Table 1).

### Program objective 4. Encourage self-identification as a scientist

Students who self-identify as scientists are more likely to apply to graduate school and continue on an academic path [35]. Science identity is influenced by multiple external and internal factors, including competence in scientific knowledge and skills, and demonstration of scientific competence to other members of the scientific community [41]. The BIG Summer program includes elements designed to foster science identity by targeting each of these contributing factors. The opportunity to work with a team on a research project provides students with continuous affirmation and recognition of science identity by members of the scientific community. The QCBio workshops instill confidence in the student’s scientific competence through hands-on mentoring in inquiry-driven scientific research. Toward the end of the program, writing an abstract and presenting a poster to the larger scientific community gives students the opportunity to demonstrate their scientific competence by communicating their findings. Students also attend weekly QCBio internal research lunches. Finally, the program provides mentorship opportunities from day-to-day mentors and UCLA faculty. Mentorship from individuals with shared attitudes, beliefs, outlooks, and values has been shown to increase commitment to STEM fields [42]. To achieve this goal, the BIG Summer program provides students with day-to-day mentors and faculty mentors who are relatable, which is expected to strengthen students’ self-identification as scientists.

### Program objective 5. Provide mentorship and professional development

To foster continuity in the quantitative biology training pipeline, BIG Summer provides students with structured programs for career development (Fig 2). Summer Programs for Undergraduate Research (SPUR), an umbrella program for all undergraduate summer programs at UCLA, holds professional development activities for undergraduates. Students in the program attend relevant sessions such as “How to Write a Research Paper in the Sciences,” “Applying to the PhD,” and “Creating Scientific Abstracts and Posters.” QCBio hosts an information session that provides information about relevant graduate degree programs at UCLA. Students can also choose to take an optional GRE preparatory class that meets after working hours. All students are guided in the process of designing Individual Development Plans, and are required to create a plan and discuss it with a mentor during the summer.

After the summer ends, faculty mentors are encouraged to continue their relationships with their students, and students are encouraged to apply for conferences and graduate school. Students often continue to be engaged in their projects or in the PI’s other research. Many students contribute to manuscripts for publication. QCBio provides funding for students to present their research at national conferences.

To assess the impact of the BIG Summer Program on the students’ career development, we asked whether participation in the BIG Summer Program affected their interest in attending graduate school. Two-thirds of participants responded that the program inspired them to attend graduate school (Table 1) and choose the right graduate program (Table 1). Of the 70 students who responded to the survey, 62 reported that the program had helped them to make connections that were useful for their careers (Table 1). Sixty-four of the 70 students had recommended BIG Summer to other students (Table 1).

### Program objective 6. Inspire students to continue their education in quantitative biology

Previous studies report that educational programs that engage students in inquiry-driven research, encourage self-identification as a scientist, and provide mentorship and professional development, can achieve outcomes that include increased attendance at graduate school [43–48]. As the BIG Summer program was developed to specifically increase student self-efficacy, scientific identity, and internalization of scientific values, we hypothesized that participation in the BIG Summer program would result in increased scientific integration as evidenced by a higher fraction of students attending graduate school in related fields. To test our hypothesis and gain a greater understanding of the impact of the BIG Summer program on student outcomes, we determined through surveys and web-based searches whether alumni had entered graduate school, and if so, the degree and field of study. We were able to obtain career data on approximately 85% of our participants (Table 2). Among students who attended BIG Summer, 75% (69/92, 65–83% 95% confidence interval, Wald test) of the students who had received their bachelor’s degree and for whom we have career data were enrolled in graduate school (Table 2). Among BIG Summer participants, 76% (62–86%) of women had enrolled in a post baccalaureate program (Table 2). Among BIG Summer alumni, 69% (51–83%) of URM students (including Black, Hispanic and Native American students) had enrolled in post-graduate education programs (Table 2). For the 12 Black students for whom we have data, six were enrolled in PhD or MD/PhD programs, two were enrolled in MD programs and four were pursuing other graduate degrees. Among all BIG Summer alumni, 14% (8–22%) were enrolled in computer science, math or statistics graduate school (Table 2). Among women participants in BIG Summer, 16% were in graduate school for computer science, mathematics or statistics.

**Table 2:** Outcomes for BIG Summer participants and a control group of nonparticipants.

	Participants	95% confidence intervals, Wald test	Control Group	95% confidence intervals, Wald test
All				
Total students	111		72	
Students with followup data	95		58	
Students with followup data who completed their bachelor’s degree	92		58	
Graduate school	69		34	
% Graduate school	75%	65–83%	59%	46–70%
PhD (including MD/PhD)	43		20	
%PhD out of students with data and bachelor’s degree	47%	37–57%	34%	24–47%
Graduate school in Bioinformatics or related field	28		11	
%Graduate school in Bioinformatics or related field out of all students with bachelor’s and data	30%	21–39%	20%	11–31%
Graduate school in Biology or related field	13		7	
%Graduate School in Biology or related field	14%	8–22%	11%	5–19%
Graduate school in computer science, math, statistics	13		4	
% Graduate school in computer science, math, statistics	14%	8–22%	6.9%	2–17%
Graduate School in Bionformatics, biology, computer science, math, or statistics	54		22	
% Graduate school in Bionformatics, biology, computer science, math, or statistics	59%	47–66%	38%	26–51%
Employed	27		24	
% Employed out of students with bachelor’s and data	29%	20–38%	41%	30–54%
Employed in Related Field	22		15	
% Employed in Related Field out of employed	81%	63–92%	63%	43–79%
Underrepresented minorities				
Total students	38		19	
Students with followup data	30		16	
Students with followup data who completed their bachelor’s degree	29		16	
Graduate school	20		12	
% Graduate school	69%	51–83%	75%	50–90%
PhD (including MD/PhD)	12		6	
%PhD out of students with data and bachelor’s degree	41%	25–59%	38%	18–61%
Graduate school in Bioinformatics or related field	8		3	
%Graduate school in Bioinformatics or related field	28%	15–46%	19%	6–44%
Graduate school in Biology or related field	6		4	
%Graduate school in Biology or related field	21%	9–39%	25%	9–50%
Graduate school in computer science, math, statistics	1		2	
%Graduate school in computer science, math, statistics	3.4%	.001–19%	13%	2–37%
Graduate school in Bionformatics, biology, computer science, math, or statistics	15		9	
% Graduate school in Bionformatics, biology, computer science, math, or statistics	52%	34–69%	56%	33–77%
Employed	9		4	
% Employed out of students with bachelor’s and data	31%	17–49	25%	10–50%
Employed in Related Field	6		1	
% Employed in Related Field out of employed	67%	35–88%	25%	3–71%
Women				
Total students	60		41	
Students with followup data	51		32	
Students with followup data who completed their bachelor’s degree	50		32	
Graduate school	38		14	
% Graduate school	76%	62–86%	44%	28–61%
PhD (including MD/PhD)	29		8	
%PhD out of students with data and bachelor’s degree	57%	44–71%	25%	13–42%
Graduate school in Bioinformatics or related field	16		3	
%Graduate school in Bioinformatics or related field	32%	21–46%	9.4%	2–25%
Graduate school in Biology or related field	10		3	
%Graduate school in Biology or related field	20%	11–33%	9.4%	2–25%
Graduate school in computer science, math, statistics	8		2	
%Graduate school in computer science, math, statistics	16%	8–29%	6.3%	0.7–21%
Graduate school in Bionformatics, biology, computer science, math, or statistics	34		8	
%Graduate school in Bionformatics, biology, computer science, math, or statistics	68%	54–79%	25%	13–42%
Employed	12		18	
% Employed out of students with bachelor’s and data	24%	14–38%	56%	39–72%
Employed in Related Field	8		11	
% Employed in Related Field out of employed	67%	39–86%	61%	38–80%

Due to the nature of the BIG Summer program and the intensive time commitment required from participants, it is likely that there is a degree of self-selection for participants who are inclined to attend graduate school. To disentangle the impact of participation in BIG Summer on its participants, we used two approaches. First, we compared the outcome for BIG Summer with other similar programs. A study of the Meyerhoff Scholars Program reports that almost half of the students went on to STEM graduate school [49]. Outcomes for BIG Summer alumni are even stronger than for Meyerhoff Program alumni. As another example, for The Leadership Alliance, another program intended to prepare undergraduates for degrees in STEM, an average of 42% of students enrolled in PhD programs, with two-thirds in STEM disciplines [50]. The rate for BIG Summer alumni compares favorably to this program as well.

As a second approach to evaluate the impact of BIG Summer on its participants, we identified a control group of students who applied to BIG Summer and were accepted, but chose not to attend. The GPA’s for the participant (3.6) and control (3.7) groups were not statistically significantly different based on Student’s t-test. The fraction that are women (41/72 or 57% for the control group, 60/111 or 54% for the participant group) is not significantly different by chi-square analysis. The fraction that are URM students (19/72 or 26% for the control group, 38/111 or 34% for the participant group) is not significantly different by chi-square test (Table 2). Among the students in the control group, 26% were in the 2016 cohort, 34% were in the 2017 cohort, and 40% were in the 2018 cohort. Among students in the participant group, 29% were in the 2016 cohort, 32% were in the 2017 cohort, and 39% were in the 2018 cohort. The fraction of students in the control and participant groups in different BIG Summer cohorts is not significantly different by chi-square test. Most of the participating students were rising seniors or rising juniors (Table 3). The distribution of year in college for the control and participant students is not significantly different by chi-square test.

**Table 3:** A. Distribution of controls and participants by year in college. B. Distribution of URM controls and URM participants by year in college.

**A**		
	Controls	Participants
Rising Freshman	0	1
Rising Sophomore	2	5
Rising Junior	15	31
Rising Senior	29	46
Participated in same calendar year as graduation	4	10
Participated 1 calendar year after graduation	2	2
Participated 2 calendar years after graduation	1	0
Unknown	5	0
Total	58	95
Average years (BIG Summer–Graduation Year)	1.1	1.3
**B**		
Rising Freshman	0	0
Rising Sophomore	0	3
Rising Junior	3	7
Rising Senior	8	14
Participated in same calendar year as graduation	2	5
Participated 1 calendar year after graduation	2	1
Participated 2 calendar years after graduation	1	0
Unknown	0	0
Total	16	30
Average years (BIG Summer–Graduation Year)	0.6	1.2

**Fig 5:**
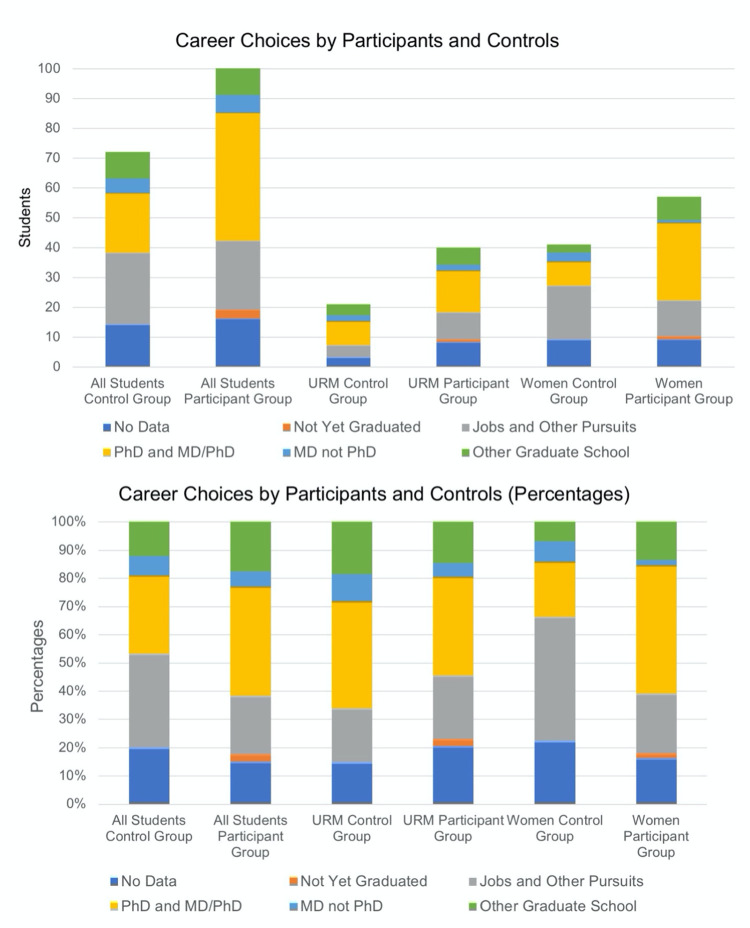
Career choices by participants and controls. Surveys and web-based searches were used to determine the postgraduate plans of BIG Summer participants and a control group composed of students who applied to the BIG Summer program were accepted but did not matriculate. (A) The following data are plotted: the number of students for whom we do not have data, the number of students who have not yet graduated, the number of students enrolled in a PhD or MD/PhD program, the number of students in an MD program, the number of students in another type of graduate school, and the number of students who are employed or engaged in other pursuits. These data are provided for all participants, URM participants and women participants. (B) The same data as in A are shown as percentage of participants.

Among the participants, 75% (95% confidence interval 65%-83%) (69/92) of those who had completed their bachelor’s degree and for whom we had data on their post-graduate plans chose to continue to graduate school (Table 2). Our rubric for assigning students to different outcome categories is provided as Supplementary Information Document 4 in S1 File. Among the control group, 59% (46%-70%) (34/58) of those who had graduated and for whom we have data chose to attend graduate school (Table 2). Within the participant group, 43 students had entered a PhD program (47%, 37–57%), while 20 of the students in the control group had entered a PhD program (34%, 24–47%) (Table 2, Fig 5). Among students from groups underrepresented in biosciences, 20 out of 29 who graduated and for whom we have information (69%, 51–83%) continued to graduate school, while in the control group, 12 out of 16 (75%, 50–90%) continued to graduate school (Table 2, Fig 5). 12 of the 29 URM students in the participant group entered a PhD program (41%, 25–59%), while 6 of the URM students in the control group entered a PhD program (38%, 18–61%) (Table 2, Fig 5). For URM students, the number of students is small, which makes it difficult to determine whether the program had a significant impact. Also, the control group of students who chose to attend a different summer program may have received a similar experience at the program they attended.

Among women students, 38 out of 50 BIG Summer students who completed their bachelor’s degree and for whom we have information, continued to graduate school (76%, 62–85%) (Table 2, Fig 5). For the control group, 14 out of 32 students entered graduate school (44%, 28–61%) (Table 2, Fig 5). Among women BIG Summer participants, 29 (57%, 44–71%, Table 2) entered a PhD program, while 8 of the women in the control group had begun a PhD program (25%, 13–42%) (Table 2, Fig 5).

**Fig 6:**
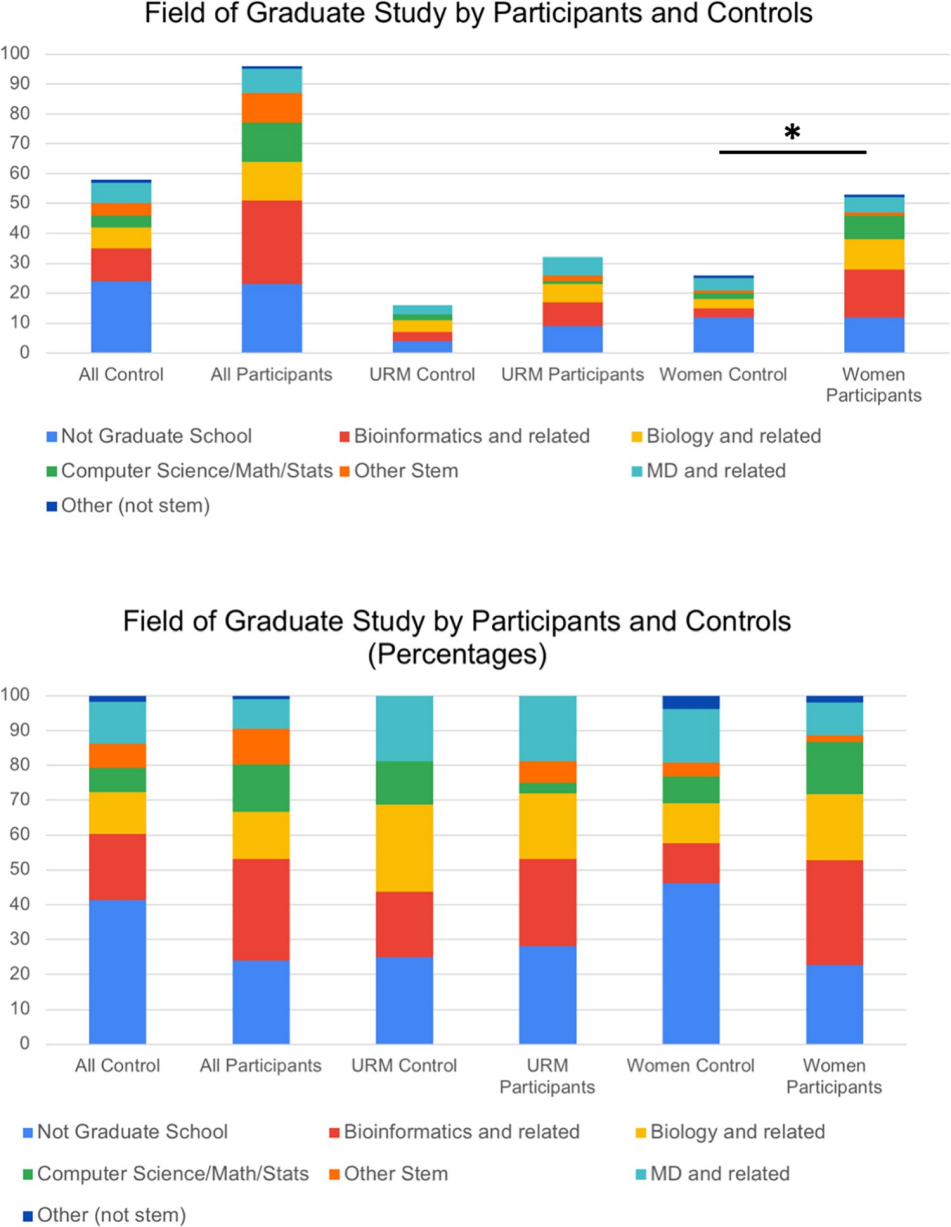
Comparison of fields of study for BIG Summer participants and controls in graduate school. Survey and web-based searches were used to determine the field of study for participants and controls that attended graduate school. Data are shown for all students (A), underrepresented minorities (B), and women (C). Among women, there is a significant increase in the number of students who attended graduate school in bioinformatics, biology, math, computer science, statistics or related fields among BIG Summer participants compared with the control group (uncorrected chi-square p = 0.024).

We investigated in more detail the field of study for students who went on to graduate work. Among all BIG Summer alumni, 30% (21–39%) continued to graduate school in bioinformatics, 14% (8–22%) continued in biology and related fields, and 14% (8–22%) continued in computer science, math or statistics (Table 2, Fig 6). Among the students in the control group, 20% (11–31%) continued in bioinformatics, 11% (5–19%) in biology and related fields, and 6.9% (2–17%) in computer science, math and statistics (Table 2, Fig 6). Among the URM participants, 28 (15–46%) students continued in bioinformatics, 21% (9–39%) in biology and 3.4% (0.001–19%) in computer science, math and statistics (Fig 6). In the control group, 19% (6–44%) of URM students continued in bioinformatics, 25% (9–50%) in biology and related fields, and 13% (2–37%) in computer science, math and statistics (Table 2, Fig 6). Among the women in the participant group, 32% (21–46%) entered graduate school in bioinformatics and related fields, 20% (11–33%) entered graduate school in biology and related fields, and 16% (8–29%) entered graduate school in computer science, math and statistics (Table 2, Fig 6). For women in the control group, 9.4% (2–25%) entered graduate school in bioinformatics or a related field, 9.4% (2–25%) entered graduate school in biology or a related field, and 6.3% (0.7–21%) entered graduate school in computer science, math or statistics. We tested our null hypothesis that students who participated in the BIG Summer program were more likely to enter graduate school in a field related to bioinformatics by comparing these values for the control (expected) and participant (observed) groups. For women, the number that entered bioinformatics, biology, computer science or related fields was significantly higher for students who participated in the program compared with controls (uncorrected chi-square, *p* = 0.024). While our primary hypothesis that participation in BIG Summer would result in more women entering a PhD program was not statistically significant, this secondary hypothesis, that participation in BIG Summer would result in more women entering a PhD program in a related field was significant. These findings are hypothesis generating and can be tested further in future studies.

**Fig 7:**
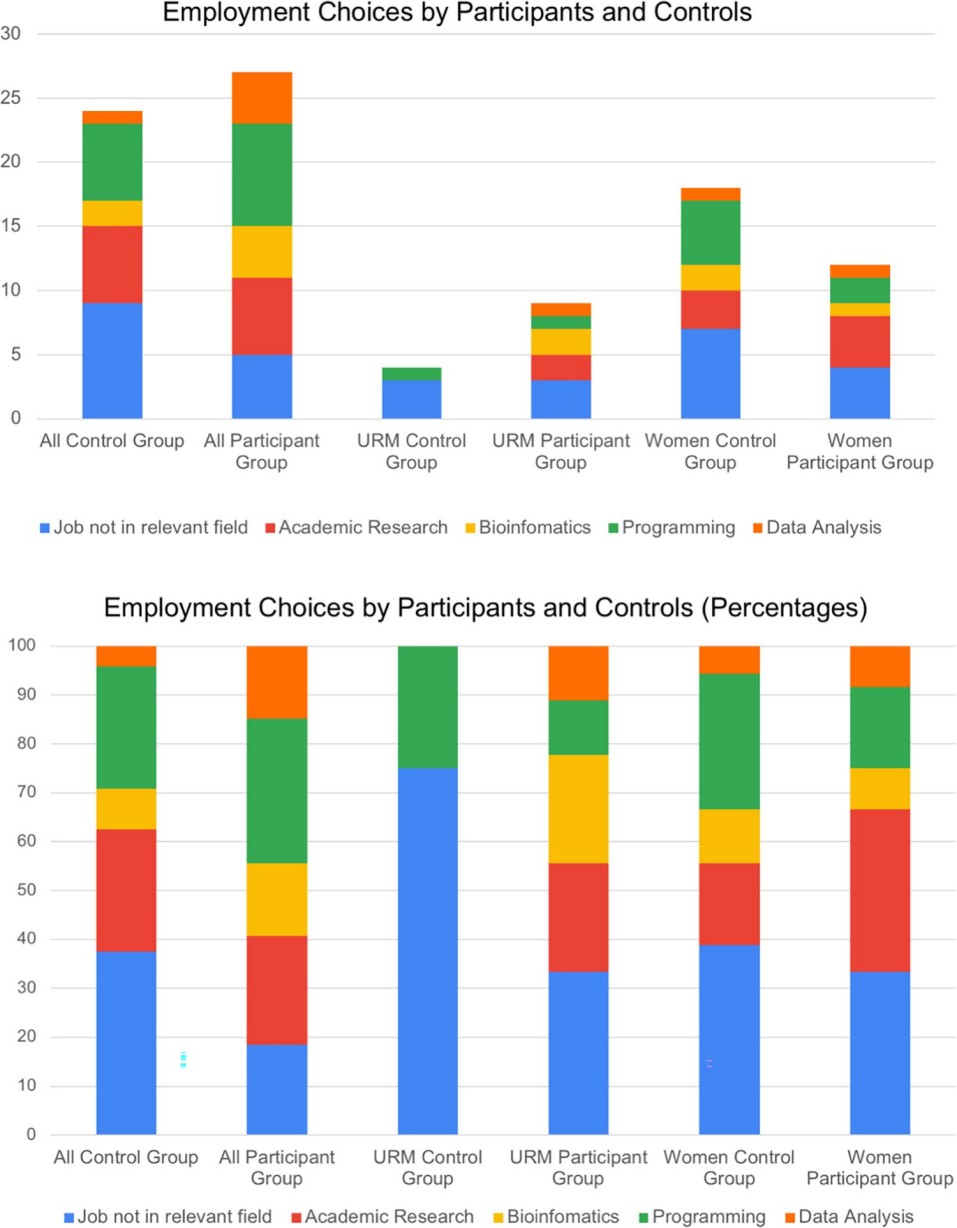
Comparison of types of jobs pursued by BIG Summer participants and controls. Surveys and web-based searches were used to determine the types of jobs held by BIG Summer alumni and controls. Data are shown for all students (A), underrepresented minorities (B), and women (C).

Finally, we investigated the types of jobs that the BIG Summer alumni and controls pursued after graduation. Among the 27 students with jobs in the participant group, 6 (22%) were involved in academic research, 4 (15%) were involved in bioinformatics, 8 (30%) were employed in programming, and 4 (15%) held jobs associated with data analysis (Fig 7). For BIG Summer alumni, 81% (63–92%) of those who were employed, were in a related field (Table 2). For students in the control group who held jobs, 63% (43–79%) were employed in a related field (Table 2). For URM students, 6 program alumni had jobs in related fields out of 9 (67%) students with jobs, while in the control group, 1 of 4 (25%) employed individuals in the group held a job in a related field (Table 2, Fig 7). Among women, 8 BIG Summer alumni had jobs in a related field out of 12 employed program alumni (67%) (Table 2, Fig 7). In the control group, 11 women had jobs in a related field out of 18 women who were employed (61%) (Table 2, Fig 7).

To further assess the effect of BIG Summer on its participants, we performed a logistic regression analysis with the data from the control and participants groups and information about whether they are enrolled in a Ph.D. program. The logistic regression analysis allowed us to test our hypothesis that the likelihood that a student attended graduate schools was different for students who attended the BIG Summer program compared with controls. We fit a logistic regression model and discovered that the Group (Control or Participant) by woman (Male or Female) interaction term had a statistically significant coefficient (*p = 0*.*005*) at α = 0.05 for predicting likelihood of obtaining a Ph.D., suggesting that for women, participation in BIG Summer affected whether or not they enrolled in a PhD or MD/PhD program (Table 4).

**Table 4:** Logistic regression results.

	Coefficient	Standard error	z	P>/z/	[0.025	0.975]
Group	-0.5212	0.56	-0.931	0.352	-1.619	0.576
Female	-0.727	0.578	-1.257	0.209	-1.861	0.407
URM	0.4861	0.732	0.664	0.507	-0.95	1.922
Group * Female	1.9298	0.697	2.784	0.005	0.574	3.305
Group * URM	-0.3966	0.766	-0.518	0.605	-1.897	1.104
Female * URM	-0.545	0.733	-0.743	0.457	-1.982	0.892

Model: PhD ~ Group + Female + URM + Group * Female + Group * URM + Female * URM

### Funding sources for Bruins-in-Genomics Summer Program

The BIG Summer program is supported by grants from the University of California Office of the President 2014–15, the National Science Foundation 1758002, the National Institutes of Neurological Disease and Stroke R25NS115554 and the National Institute of Mental Health R25MH109172. Institutional funds from the QCBio, the David Geffen School of Medicine and the UCLA Graduate Division also support the program. The University of California Office of the President (UCOP) provided funding for students from Historically Black Colleges and Universities. This grant supported partnerships with Fisk University, Florida A&M, and Spelman. The National Science Foundation (NSF) Research Experiences for Undergraduates (REU) program included the program on its website, increasing visibility. The NSF program is focused on supporting students from diverse backgrounds, students who are the first generation to attend college, students with disabilities, and students from schools that do not have a PhD program in bioinformatics.

## Discussion

Undergraduate research experiences and faculty mentorship are important for encouraging students with an interest in STEM to continue their education and remain in STEM-related fields. Previous studies have shown that students are more likely to persist in the sciences if they identify as a scientist, feel that the work is aligned with their values, and have a sense of scientific efficacy [23, 31, 43, 51]. BIG Summer seeks to create an environment in which these conditions are met through a combination of didactic skill development, research experiences, community development, mentorship, and professional guidance designed to increase student self-efficacy, science identity, and scientific value integration.

In the first five years of BIG Summer, the program developed a national reputation resulting in a strong and large application pool. Alumni who responded to our follow-up survey overwhelmingly reported that their participation in the program provided skills that were valuable for their career, influenced their research interests, and introduced them to the new scientific areas. Alumni reported that participation in the program allowed them to make connections that were useful in their careers, inspired them to go to graduate school, and helped them select the right graduate program. We compared the post-graduate career choices of students who attended BIG Summer with a control group of students who applied to BIG Summer and were accepted but chose another summer plan. Women who participated were more likely than women in the control group to attend graduate school, supporting the hypothesis that participating in BIG Summer increased the likelihood that women participants would continue to graduate school. Fully 30% of BIG Summer participants, and 20% of the control group, elected to continue with postgraduate education in bioinformatics or a closely related field. The percentage of students in graduate school for Bioinformatics and related fields was similar for students from underrepresented backgrounds (28% for BIG Summer participants and 19% of the control group) and for women (32% for BIG Summer participants and 9.4% of the control group). Of the BIG Summer students who had secured employment, a high fraction was employed in a field related to bioinformatics (81%, 62–92%). In the control group, 63% (43–79%) were employed in bioinformatics or a similar field.

Our data did not reveal an increase in the fraction of URM BIG Summer students who entered PhD or MD/PhD programs compared with our control group of students who had also taken the initiative to apply to BIG Summer, were accepted, but declined. A much larger study revealed that participation in the four-year spanning Meyerhoff Program was associated with an increased fraction of students entering a PhD program in a STEM field, with the control being students who were invited to participate in the Meyerhoff Program but did not [22]. Whether our 8-week program increases matriculation in graduate education programs over similarly self-selected control group currently remains unknown, largely because of a number of limitations of the present study. The numbers of students in the participant and control groups are small because the program has only been in existence for a few years. It is possible that women and men decline an offer to participate in the program for different reasons, which would affect our ability to draw conclusions about the impact of the program on its male and female participants. Further, among URM students, the control group was already closer to graduation (and their pending enrollment in a graduate program may be a reason for them declining BIG Summer offer) than the participant group (Table 3B). Over time, as participant and control student numbers increase, and students in past years graduate from college, complete their gap year if taken, and enter graduate school, the impact of the program on its participants may become clearer. Future surveys will also provide information on the persistence of program goals.

We believe that other institutions can develop programs similar to the Bruins-in-Genomics Program and offer some advice based on our observations. First, we suggest advertising the program at conferences such as ABRCMS and SACNAS. Students presenting posters and winning awards at such conferences were effective methods for advertising the BIG Summer program. Second, we highly recommend an application for the program that asks questions about the applicants’ courses, their research experience, their research interests, and their contributions to diversity. The BIG Summer application form is included as Supplementary Information Document 5 in S1 File. Third, we encourage institutions hosting programs to provide student participants with training at the beginning of the program so that the students have tangible skills that they can bring to their research. Fourth, we encourage training for daily mentors prior to the program on developing realistic projects and aligning expectations. We also encourage training for daily mentors about culturally-aware mentorship. Fifth, we encourage institutions to develop regular check-in mechanisms with the students and the mentors. Our program has instituted google form check-ins on weeks 1, 2, and 4 to identify problems with mentor-student pairings that need to be addressed. Sixth, we encourage institutions replicating our program to ensure there are mechanisms for cohort-building among the students. BIG Summer organizes weekly student-led journal clubs as well as optional social events for participants. Seventh, we encourage institutions to provide access to professional development opportunities for the students. BIG Summer provides access to GRE preparation sessions and organized profession development sessions that help students develop tangible skills such as presenting a poster, writing an abstract, and writing a resume. Eighth, we encourage other institutions seeking to host similar programs to provide the students with a capstone experience such as a written abstract and an oral poster presentation in a poster session. Finally, we suggest stressing post-program engagement, for instance, support for students to prepare posters and present them at conferences such as ABRCMS. We believe that all of these strategies contributed to the program’s success.

Overall, students who attended BIG Summer overwhelmingly reported that they had learned skills that were valuable for their careers or studies. For women, who are nationally less likely to enter graduate school in quantitative fields, we observed an increased likelihood that the participants would continue to graduate school in a related field than student in a control group. These results attest to the effectiveness of our program in addressing the need for an increased and more diverse pool of STEM-trained individuals.

## Supporting information

S1 Data (XLSX)

S1 File (PDF)
