# Supplementary material for: Bruins-in-Genomics: Evaluation of the impact of a UCLA undergraduate summer program in computational biology on participating students
Source: PLoS One. 2022 May 27;17(5):e0268861. doi: 10.1371/journal.pone.0268861 (PMC9140266; doi:10.1371/journal.pone.0268861)
Supplement: S1 File — (PDF) [file pone.0268861.s002.pdf]

Supplementary Information

Supplementary Document 1: Survey instrument administered to program alumni via Google forms to BIG Summer program alumni.

Alumni Survey: Bruins-in-Genomics Program

Thank you for completing this survey! The results of this survey are important for all of our future grant proposals, as well as for reports to the funding agencies. If you received this survey in error, please reply to my email so that we can correct our records. We thank you again for taking the time to respond.

* Required

First Name *

Your answer

Last Name *

Your answer

Your preferred email address: *

Your answer

What year did you participate in BIG Summer? *

- ○ 2015
- ○ 2016
- ○ 2017
- ○ 2018
- ○ 2019

Did you attend a scientific conference in the year following BIG Summer? Check all that apply.

- ☐ ABRCMS
- ☐ SACNAS
- ☐ Other:

Did your research result in a publication?

- ○ Yes
- ○ Working on it.
- ○ No

If yes, please include the citation below. If it is in progress, what is the status?

Your answer

What year did you finish (or will you finish) your bachelor's degree? *

- ◯ 2016
- ◯ 2017
- ◯ 2018
- ◯ 2019
- ◯ 2020
- ◯ 2021 (Expected.)
- ◯ 2022 (Expected.)

What did you do (or will you do) after finishing your bachelor's degree?

- ◯ Attend graduate or professional school.
- ◯ Start a career in private or public sector.
- ◯ Not sure/still considering options.
- ◯ Other:

If you started graduate or professional school, in what subject or field?

Your answer

What institution?

Your answer

What degree goal?

- ○ Master's Degree
- ○ PhD
- ○ MD
- ○ Other:

If you chose to start your career, please briefly describe your job and the organization.

Your answer

Is there anything else you'd like to share with us about your career plans or educational goals? *

Your answer

Tell us about your other achievements (awards, honors, etc.) since BIG Summer.

Your answer

Have you recommended BIG Summer to other students? *

- ○ Yes
- ○ No

How did BIG Summer impact you? *

	Yes	No	I don't know
Influenced my research interests.	○	○	○
Introduced me to new scientific areas.	○	○	○
Inspired me to go to graduate school.	○	○	○
Helped me choose the right graduate program.	○	○	○
Made connections that are useful to my career.	○	○	○
Taught me skills that are useful to my career and/or studies.	○	○	○

Please share your thoughts on the value or impact of BIG Summer. *

Your answer

Submit

Never submit passwords through Google Forms.

This content is neither created nor endorsed by Google. Report Abuse - Terms of Service - Privacy Policy

Google Forms

Supplementary Document 2: Information included in the data abstraction tool for monitoring career paths for students who participated in BIG Summer and controls.

Year of Program
Last Name
First Name
Citizenship
Ethnicity
First Generation College
Gender
Undergraduate University
Major
UCLA PI/Lab
Grant
Email address
Preferred or alternative email
Conferences attended
Linked in Profile
Responded to survey
Year of BS/BA
Graduate Field of Study
Graduate University
Graduate Program (degree)
Start Date
Year of Graduate Degree
Jobs of Notes
Plans/Other Notes
Last updated

Supplementary Document 3. Student learning objectives for the BIG Summer program.

A. To develop competence with computer programming skills.

B. To develop competence with skills needed to process, and analyze next generation sequencing data.

C. To develop skills to critically read, interpret and evaluate primary papers in the quantitative biology literature.

D. To develop research skills through inquiry-driven research projects in individual laboratories.

E: To develop skills communicating research findings in written and oral formats.

Supplementary Document 4. Rubric for Assigning Students to Categories for Student Outcomes

Not Graduate School: Students who did not attend graduate school.

Bioinformatics and related: Students who attended graduate school and studied bioinformatics or a bioinformatics-related field (for example, computational biology, computational genomics, etc.).

Biology and related: Students who attended graduate school and studied biology or a biology-related field (for example, cellular biology, molecular biology, etc.).

Computer Science/Math/Stats: Students who attended graduate school and studied computer science, mathematics or statistics.

Other STEM: Students who attended graduate school and studied a different STEM field (for example, physics).

MD and related: Students who attended graduate school and studied medicine or a medical-related field (for example, dentistry).

Other (not stem): Students who attended graduate school and did not study a STEM field.

Supplementary Document 5. BIG Summer application form.

UCLA Institute for Quantitative & Computational Biosciences

B.I.G. Summer Application

Application portal for B.I.G. Summer 2021 is now open! Admission is competitive and early applications are strongly encouraged.

PROGRAM DATES: **JUNE 21 TO AUGUST 13, 2021**

APPLICATION DEADLINE FOR EARLY DECISION: **January 4, 2021 at 5:00PM PST**

FINAL DEADLINE: **March 1, 2021 at 5:00PM PST**

Applicants must be:

- a U.S. citizen, permanent resident, or F-1 visa holder;
- a rising junior or senior;
- have a GPA of 3.0 or higher;
- have some familiarity with at least one programming language (e.g. python, pearl, R, Java, MAT-LAB, C++, etc.);
- preferably have taken bioinformatics or biostatistics courses.

A finalized application will include:

- a complete online application form;
- one letter of recommendation from a faculty member;
- one copy of your unofficial transcript;

Search

- one document containing short responses to a set of eight questions posted.

Questions? Contact BIGSummer@ucla.edu.

APPLICATION INSTRUCTIONS

1. Complete online form, below
2. Upload with the online form: one copy of your unofficial transcript
3. Upload with the online form: one document containing responses to the following questions:
   - **Question 1:** Describe your experience with computer programming (e.g. python, pearl, R, Java, MAT-LAB, C++, etc.) and self-evaluate level of proficiency for each language(100 word max).
   - **Question 2:** Describe the computer science courses you have taken or plan to take (100 word max).
   - **Question 3:** Describe the biology science courses you have taken or plan to take (100 word max).
   - **Question 4:** Describe any prior research experience you have had (100 word max).
   - **Question 5:** Describe your interest in research and your educational goals (100 words max).
   - **Question 6:** Describe why you are interested in this program (100 word max).
   - **Question 7:** List three UCLA faculty whose research you are interested in and describe why (up to 100 words each). Potential B.I.G. Summer mentors can be found on our partner programs' websites:
   - UCLA Institute for Quantitative and Computational Biosciences (QCBio)

- UCLA Interdepartmental Graduate Program (IDP) in Bioinformatics
- UCLA Genetics and Genomics BioSciences Program
- Gene Regulation, Epigenomics and Transcriptomics @ UCLA Molecular Biology Institute
- UCLA Dept. of Ecology and Evolutionary Biology
- UCLA School of Dentistry Research Centers

- **Question 8:** UCLA QCB is committed to attracting exceptional students who will contribute to the University's diversity. Describe how you have or may contribute to diversity (500 word max).

4. Arrange for one faculty member (e.g. research mentor, class professor) to submit a letter of recommendation. **An automated email will be sent directly to your referee** with directions on how to upload your letter, once your application has been submitted.

Fields marked with an * are required

First Name *

Last Name *

Email address *

Phone

Gender *

- [ ] Female
- [ ] Male
- [ ] Decline to state

Race/Ethnicity * ☐

- [ ] AI = American Indian/Alaska Native
- [ ] AA = African-American/Black
- [ ] MA = Mexican/Mexican-American/Chicano
- [ ] OS = Other Hispanic/Latino (includes Cuban, Puerto Rican, Central and South American)
- [ ] FA = Filipino/Filipino-American
- [ ] PI = Pacific Islander (includes Micronesian, Polynesian, other Pacific Islanders)
- [ ] CA = Chinese/Chinese-American
- [ ] EI = East Indian/Pakistani
- [ ] JA = Japanese/Japanese-American
- [ ] KA = Korean/Korean-America
- [ ] VA = Vietnamese/Vietnamese-American
- [ ] OA = Other Asian (not including Middle Eastern)
- [ ] WH = White/Caucasian
- [ ] OT = Other (Please specify)
- [ ] DS = Decline to State

Are you a first generation college student (neither parent has a higher degree than high school diploma)? *

- [ ] Yes
- [ ] No
- [ ] Decline to state

Are you a person with a disability (physical or mental impairment that substantially limits one or more major life activities)? *

- [ ] Yes
- [ ] No
- [ ] Decline to state

Country of Citizenship *

United States

Please select your US status: * ☐

- [ ] U.S. Citizen
- [ ] Permanent Resident
- [ ] XX Visa
- [ ] Not a resident of the U.S.

Name of Undergraduate Institution *

Does your University/College offer a PhD Program in Bioinformatics, Genomics or Computational Biology? *

- [ ] Yes
- [ ] No
- [ ] Decline to state

Provide the month/year of BA/BS degree: *

In the fall of 2021, I will be a: *

- [ ] Sophomore
- [ ] Junior

- [ ] Senior
- [ ] Other: explain

Undergraduate Major *

Current Cumulative GPA *

Are you interested in (check one box): *

- [ ] On-campus
- [ ] Remote/online
- [ ] Either/No preference

Are you interested in...(check all that apply) *

- [ ] Bioinformatics (BI)
- [ ] Medical Informatics (MI)
- [ ] Systems Biology (SB)
- [ ] Neuroscience (NS)
- [ ] Dental, Oral and Craniofacial Research (DOC)

Are you a UCLA student? *

- ○ Yes
- ○ No

Provide the first name of your referee: *

Provide the last name of your referee *

Provide the title or position of your referee: *

Provide the email address of your referee *

Resume * ☐

Select Files

Unofficial Transcript * ☐

Select Files

Responses to questions 1 - 8 * ☐

Select Files

Submit

UCLA QCB BOYER HALL

© Copyright 2021 UCLA - Login
